# Automated segmentation of blood cells in Giemsa stained digitized thin blood films

**DOI:** 10.1186/1746-1596-8-S1-S37

**Published:** 2013-09-30

**Authors:** Margarita Walliander, Riku Turkki, Nina Linder, Mikael Lundin, Juho Konsti, Ville Ojansivu, Taru Meri, Ville Holmberg, Johan Lundin

**Affiliations:** 1 Institute for Molecular Medicine Finland (FiMM), Finland; 2 Center for Machine Vision Research, Department of Computer Science and Engineering, University of Oulu, Finland; 3 Haartman Institute, Finland

## Background

Assessment of erythrocytes and leucocytes in thin blood films can be used as an inexpensive diagnostic aid in a series of disease states, e.g. infections, anemia and hematological malignancies. Manual counting of cells is still considered the gold standard for example to establish the level of parasitemia in malaria. However, manual cell counting is time consuming and subject to variability [1]. We here propose an image analysis method that is a combination of adaptive histogram thresholds and morphologic characteristics for the segmentation of red blood cells (RBCs) and white blood cells (WBCs) in digitized thin blood films. The method is implemented on a virtual microscopy platform, the Webmicroscope [2].

## Methods

Ten Giemsa stained thin blood films were digitized with a microscopy slide scanner (Axio Imager Z2, Carl Zeiss MicroImaging, Jena controlled by Metafer software, MetaSystems, Altlussheim) using a 63x objective with a numerical aperture (NA) of 1.4 (Plan-Apochmat, Carl Zeiss, Jena) and oil immersion. Image acquisition was performed with a monochrome CCD camera with a 1360x1024 pixel sensor and a pixel size of 6.45 µm (CoolCube 1, MetaSystems, Altlussheim), a 1.0 camera adapter and illumination with an RGB illuminator (MetaLED Z, red 619 nanometer, green 515 nanometer, blue 465 nanometer, MetaSystems, Altlussheim). The pixel size in the digital images was approximately 0.10µm and the original TIFF images were converted into a wavelet format (Enhanced Compression Wavelet, ERDAS/Intergraph, Norcross, GA) and transferred to a virtual microscopy image server (http://fimm.webmicroscope.net/Research/Momic/tp2012) [3]. Approximately five-hundred (473 – 505) fields of view from each blood film sample were captured and stored in the database. Five of the samples were infected with *Plasmodium falciparum* and five were non-infected control samples.

**Figure 1:**
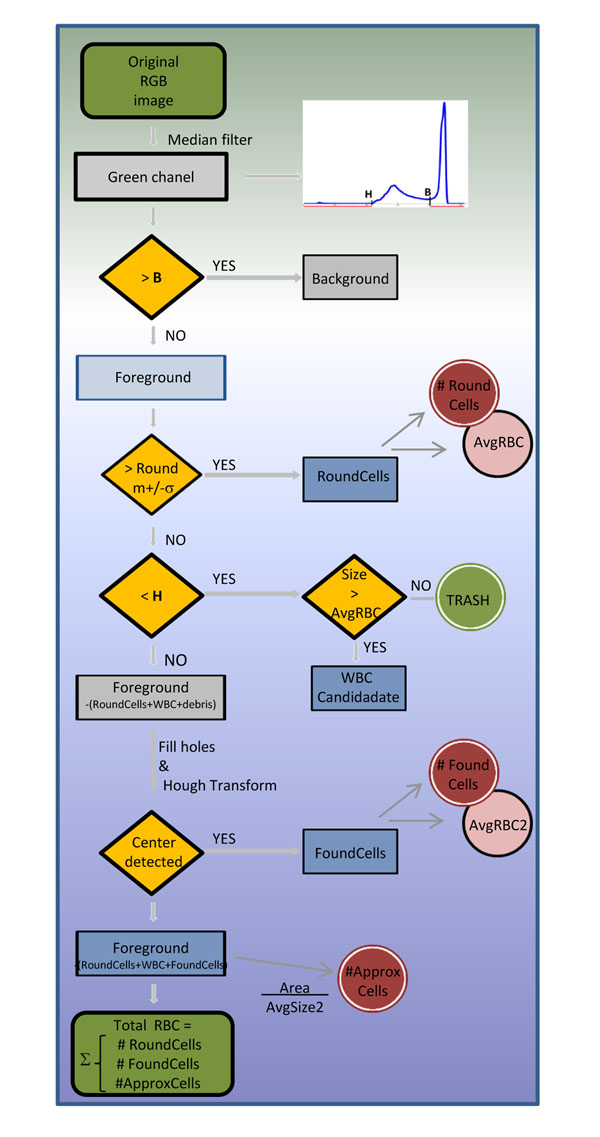
**Flowchart showing the cell segmentation process** Overview of the cell segmentation and counting process for red and white blood cells. From the original image the green channel is selected and smoothed. Dynamic thresholding allows the separation of the image in foreground and background. The foreground is split in to *RoundCells*, *WBC Candidates*, *FoundCells* and the remaining part being deformed red blood cells and clumps of red blood cells. These subimages are used to count the objects of interest i.e. red blood cells and white blood cells.

The described method (Fig. 1.) involves 1) separation of background and foreground, 2) recognition of objects that compose the foreground and 3) cell counting (i.e. RBCs and WBCs).

### Image preprocessing

As a preprocessing step for each thin blood film sample, the green channel was selected from the original RGB image [4] and smoothed by applying a median filter 3X3 to reduce the' salt and pepper' noise [5]. The green channel is extracted using a color deconvolution between the original image and a vector [0,1,0].

### Adaptive histogram thresholds

Let each pixel of the preprocessed image have intensity levels in *[0*, *1*, *2*, *…*, *L-1]* with *L*= *256*. The number of pixels with intensity level *i* is denoted by *n_i_*, *∀ i*=*0*, *1*, *2*,*…*, *L-1*, where  is the total amount of pixels. We defined the histogram distribution as *p*(*i*) = *n_i_/N*, *p*(*i*) ≥*0*, where .

**Figure 2:**
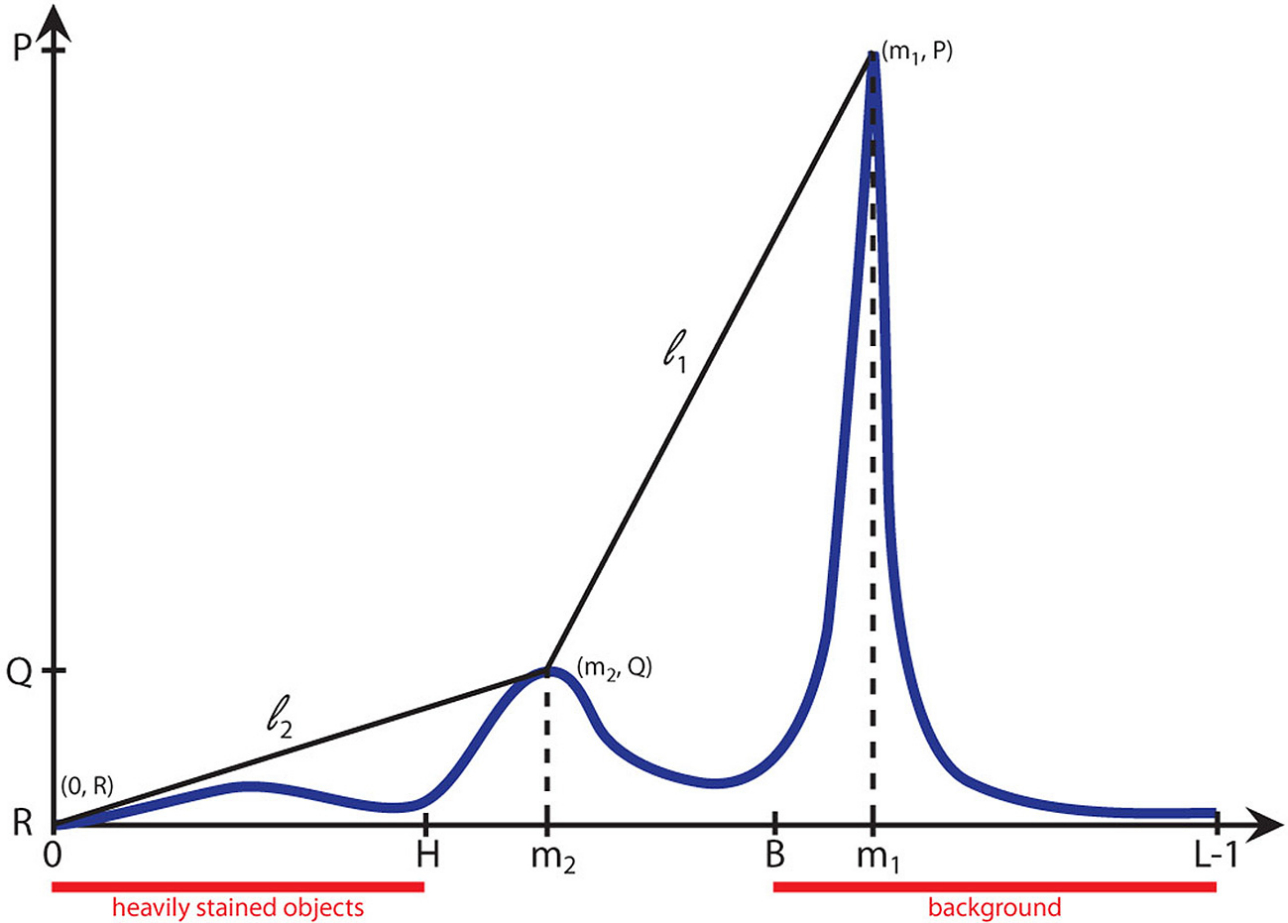
**Bimodal histogram depicting adaptive thresholds location, background intensity range and heavily stained objects range** A gray level histogram that corresponds to the green channel of a typical field of view after applying a median filter (3X3). The mode of the histogram are located at m_1_ and m_2_. B and H are the adaptive thresholds. l_1_ is a line defined between the modes of the histogram and l_2_ is a line defined between the origin of the histogram and the mode at m_1_.

For any monolayer stained blood film, the histogram is bimodal. A typical histogram shape for a monolayer thin blood film is shown in Fig. 2.

There are two local maxima located at *m_1_* and *m_2_*, where *m_2_* <*m_1_* and *P* = *p*(*m_2_*) <*p*(*m_1_*) = *Q.*

A threshold to differentiate the background from the foreground *B*, is defined by finding the maximum distance between the histogram and the line *l_1_* described between (*m_1_*, *P*) and (*m_2_*_, _*Q*) as shown in Fig. 2.

with

In a similar manner, a threshold to find heavily stained objects *H*, was defined by finding the maximum distance between the histogram distribution and the line *l_2_* described between (*0*, *R*) and (*m_1_*, *P*), with *R*= *p*(*0*);

and

The image background is given by Eq. (1), while the heavily stained objects are described by Eq. (2).(1)(2)

where = gray level Image.

### RoundCells separation

Using the image histogram and based on its bimodal shape, two important thresholds were extracted (Fig. 2). The first threshold (B) defines a binary separation between the image background and foreground. From the image foreground, all objects with roundness bigger than 0.6 were selected and the area of each of the objects was measured. The mean diameter to be 7.52µm and standard deviation of 0.06µm for the whole set of objects was calculated. Finally, only the subset of objects with an area equal to *m*+/-*σ* (3848+/-688 pixels) was chosen and defined as *RoundCells*. From this set of round objects of similar size, the average diameter was calculated and used to define a representative red blood cell, designated as *AvgRBC* (diameter ~7µm) and to establish limit diameters for WBCs (~7-21 µm) and platelets (~2-3µm).

The second threshold (H), defines the heavily stained objects in the foreground (i.e. WBC, platelets, artifacts and debris). The heavily stained objects larger than *AvgRBC* are the *FoundWBCs*.

### Detection of circular shapes by Hough transform

Hough transform is calculated on gray level images that contain only the regions of interest while the remaining is set to zero. The region of interest is composed by foreground without *RoundCells*, *FoundWBCs* and debris. The maximization of Hough transform for a radius interval  is performed, where *r* = *radius* (*AvgRBC*). The result is a set of accumulations of hits (votes). The accumulations are concentrated around the centers of the circular shapes. Hough transform detected cells are filtered by selecting the pixel with the maximum vote and deleting all the pixels with less than 20% of the votes. Thus the selection of nearly circular shapes is ensured. Finally, a morphological opening is performed to discard accumulations with less than 50 pixels. The remaining objects are centers of *FoundCells*.

### FoundCells detection

After subtracting the *RoundCells* and the heavily stained objects from the original image, to compensate for the holes left from the subtraction of the platelets, debris and parasites, a morphologic filling was performed. By using Hough transform, circular shapes were detected in the grayscale image and designated as *FoundCells*, resolving the center positions of the nearly circular objects. A second representative red blood cell *AvgRBC2* was defined from the area of *FoundCells*.

### ApproxCells detection

After subtracting the *RoundCells*, the *FoundWBCs* and the *FoundCells*, the remaining image contains fragments of RBCs and deformed RBCs which Hough transform was not able to define as circular shapes. The total area covered by these objects, named *ApproxCells* was divided by the area of *AvgRBC2* which is estimation for the number of cells that still remain without being counted *#ApproxCells*.

Finally, the total number of RBCs in the image is calculated by summing up the partial results;(3)

### **Results and discussion**

RBCs were manually annotated in 30 fields of views per thin blood film and WBCs were annotated in the entire data set (Table 1). The results from the manual counting and automated counting are shown (Table 1.) Using the annotated fields of view, automated quantification of RBCs and WBCs was compared against the manual annotations and RBCs showed an overall error rate of 0.06%, WBCs counting showed an overall error rate of 0.21%. A test for the automated counting of RBCs and WBCs was performed on whole slides of thin blood films and approximately half a million red blood cells and 477 white blood cells were counted (Table 2.). Previously published studies have addressed the separation and counting blood cells, but fixed thresholds for colors, sizes and intensity values restrict the use to particular data sets [6-10]. Here, we make use of adaptive thresholds for size and intensity values, which converges to a solution.

**Table 1:** Results comparing manual and automated cell counting

Red Blood Cells			White Blood Cells			
Sample	Annotated	Automated	Error %	Annotated	Automated	Error %

I1	3145	3160	0.476948	20	20	0

I2	4048	4058	0.247036	34	34	0

I3	2796	2782	0.500715	22	22	0

I4	2972	2958	0.471063	30	32	6.66

I5	3047	3042	0.164096	77	75	2.59

C1	3396	3389	0.206125	75	75	0

C2	3491	3482	0.257806	55	56	1.81

C3	3093	3087	0.193986	42	42	0

C4	3197	3206	0.281514	49	50	2.04

C5	3513	3514	0.028466	71	72	1.38

TOTAL	32698	32678	0.061166	476	477	0.21

%	100	99.9388	100	99.79		

Results comparing manual and automated counting for red blood cells and white blood cells (WBCs). Red blood cells were annotated in a region equivalent to 30 fields of view per film, while the annotations for WBCs were performed on 500 fields of view per film. The samples I1-I5 are *Plasmodium falciparum* infected cases, C1-C5 are non-infected controls.

**Table 2:** Results of automated red blood cell counting on whole slides of thin blood film

Cell counting					
sample	#TotalRBC	#RoundCells	#FoundCells	#ApproxCells	AvgRBC diameter μm

I1	59333	17935	40512	886	7.6804

I2	70236	16458	52600	1178	7.5457

I3	43973	23068	20475	430	7.7613

I4	46980	14237	32438	305	7.8404

I5	46090	13670	31918	502	7.5258

C1	57760	18379	38993	388	7.3918

C2	53645	23704	29669	272	6.987

C3	45462	22290	22669	503	7.5385

C4	51605	18108	32674	823	7.2068

C5	58029	16656	41098	275	7.7327

TOTALS	533113	184505	343046	5562	

Partial and total amount of red blood cells, automatic counting on 500 fields of view per sample. The first five samples are *Plasmodium falciparum* infected cases I1-I5, samples C1-C5 are non-infected controls.

## Conclusions

The segmentation of RBCs and WBCs is an easy task for a human observer. Humans have the ability of distinguishing large number of colors, shades and hues, also estimating shapes and size similarities while referring to prior knowledge, making global and local comparisons simultaneously. However, performing large scale quantification is a time consuming and tedious task.

We present an unsupervised tool for separating the foreground from the background in Giemsa stained thin blood films and an automated cell counter for RBCs and WBCs. The segmentation of blood cells in thin blood films can be used as a pre-processing step to specify the regions of interest for a secondary algorithm, e.g. the detection of malaria parasites in RBCs, morphological analysis of RBCs and WBCs and follow-up during treatment of hematological malignancies or measurement of response to chemotherapy.

## List of abbreviations

RBC: Red blood cell; WBC: White blood cell; #: Number of cells in

## Competing interests

The authors declare that they have no competing interests.

## References

[B1] O'Meara WP Barcus M Wongsrichanalai Ch Muth S Maguire JD Jordan RG Prescott WR McKenzie FE Reader technique as a source of variability in determining malaria parasite density by microscopy Malaria Journal 2006 5 118 10.1186/1475-2875-5-118 17164007 PMC1712346

[B2] Linder E Lundin M Thors C Lebbad M Winiecka-Krusnell J Helin H Leiva B Isola J Lundin J Web-based virtual microscopy for parasitology: a novel tool for education and quality assurance PLoS Negl Trop Dis 2008 2 10 e315 Epub 2008 Oct 22 10.1371/journal.pntd.0000315 18941514 PMC2565642

[B3] The WebMicroscope virtual microscopy environment http://www.webmicroscope.net/

[B4] Wermser D Haussman G E LC Segmentation of blood smears by hierarchical thresholding Computer Vision, Graphics and Image Processing 1984 25 151 168 10.1016/0734-189X(84)90100-2

[B5] Gonzalez RC Woods RE Digital Image Processing 2002 New Jersey: Prentice Hall

[B6] Kumar R Joseph DK Sreenivas TV Teager Energy based blood Cell Segmentation14th International Conference on Digital Signal Processing 2002 2 619 622

[B7] Tek FB Dempster Ag Kale I Computer vision for microscopy diagnosis of malaria Malaria Journal 2009 8 53 10.1186/1475-2875-8-153 PMC2719653 19594927

[B8] Dorini LB Minetto R Leite NJ White Blood cell segmentation using morphological operators and scale-space analysis SIBGRAPI, 2007. XX Brazilian Symposium on Computer Graphics and Image Processing 294 304

[B9] Ramesh N Dangott B Salama ME Tasdizen T Isolation and two-step classification of normal white blood cells in peripheral blood smears Journal of Pathology Informatics 2012 149 170 10.4103/2153-3539.93895 PMC3327044 22530181

[B10] Purwar Y Shah S Clarke G Almugairi A Muehllenbachs A Automated and unsupervised detection of malaria parasites in microscopic images Malaria Journal 2011 10 364 10.1186/1475-2875-10-364 22165867 PMC3254597

